# Maternal excess adiposity and serum 25-hydroxyvitamin D < 50 nmol/L are associated with elevated whole body fat mass in healthy breastfed neonates

**DOI:** 10.1186/s12884-022-04403-w

**Published:** 2022-01-29

**Authors:** Maryam Razaghi, Nathalie Gharibeh, Catherine A. Vanstone, Olusola F. Sotunde, Shu Qin Wei, Dayre McNally, Frank Rauch, Glenville Jones, Hope A. Weiler

**Affiliations:** 1 School of Human Nutrition, McGill University, Ste-Anne-de-Bellevue, Québec Canada; 2 Institut national de santé publique du Québec, Montréal, Québec Canada; 3 Department of Pediatrics, Children’s Hospital of Eastern Ontario, University of Ottawa, Ottawa, Ontario Canada; 4 Shriners Hospital for Children, Montréal, Québec Canada; 5 Department of Biomedical and Molecular Sciences, Queen’s University, Kingston, Ontario Canada; 6 Nutrition Research Division, Bureau of Nutritional Sciences, Food Directorate, Health Products and Food Branch, Health Canada, 251 Sir Frederick Banting Driveway, Room E338, Ottawa, Ontario K1A 0K9 Canada

**Keywords:** Mother-infant dyads, Body composition, Vitamin D status

## Abstract

**Background:**

Vitamin D status of pregnant women is associated with body composition of the offspring. The objective of this study was to assess whether the association between maternal vitamin D status and neonatal adiposity is modified by maternal adiposity preconception.

**Methods:**

Healthy mothers and their term appropriate weight for gestational age (AGA) infants (*n* = 142; 59% male, Greater Montreal, March 2016-2019) were studied at birth and 1 month postpartum (2-6 weeks). Newborn (24-36 h) serum was collected to measure total 25-hydroxyvitamin D [25(OH)D] (immunoassay); maternal pre-pregnancy BMI was obtained from the medical record. Anthropometry, body composition (dual-energy X-ray absorptiometry) and serum 25(OH)D were measured at 2-6 weeks postpartum in mothers and infants. Mothers were grouped into 4 categories based on their vitamin D status (sufficient 25(OH)D ≥ 50 nmol/L vs. at risk of being insufficient < 50 nmol/L) and pre-pregnancy BMI (< 25 vs. ≥25 kg/m^2^): insufficient-recommended weight (I-RW, *n* = 24); insufficient-overweight/obese (I-OW/O, *n* = 21); sufficient-recommended weight (S-RW, *n* = 69); and sufficient-overweight/obese (S-OW/O, *n* = 28). Partial correlation and linear fixed effects model were used while adjusting for covariates.

**Results:**

At birth, infant serum 25(OH)D mean concentrations were below 50 nmol/L, the cut-point for sufficiency, for both maternal pre-pregnancy BMI categories; 47.8 [95%CI: 43.8, 51.9] nmol/L if BMI < 25 kg/m^2^ and 38.1 [95%CI: 33.5, 42.7] nmol/L if BMI ≥25 kg/m^2^. Infant serum 25(OH)D concentrations at birth (*r* = 0.77; *P* < 0.0001) and 1 month (*r* = 0.59, *P* < 0.0001) were positively correlated with maternal postpartum serum 25(OH)D concentrations. Maternal serum 25(OH)D concentration was weakly correlated with maternal percent whole body fat mass (*r* = − 0.26, *P* = 0.002). Infants of mothers in I-OW/O had higher fat mass versus those of mothers in S-OW/O (914.0 [95%CI: 766.4, 1061.6] vs. 780.7 [95%CI: 659.3, 902.0] g; effect size [Hedges’ *g*: 0.42]; *P* = 0.04 adjusting for covariates) with magnitude of difference of 220.4 g or ~ 28% difference.

**Conclusions:**

Maternal and neonatal vitamin D status are positively correlated. In this study, maternal adiposity and serum 25(OH)D < 50 nmol/L are dual exposures for neonatal adiposity. These findings reinforce the importance of vitamin D supplementation early in infancy irrespective of vitamin D stores acquired in utero and maternal weight status*.*

**Supplementary Information:**

The online version contains supplementary material available at 10.1186/s12884-022-04403-w.

## Introduction

Adverse nutritional exposures in utero and in infancy impair growth [1] and increase the risk of chronic conditions later in life [2, 3]. A burgeoning body of evidence suggests that maternal-fetal transfer of vitamin D is associated with a lean body mass phenotype in childhood [4]. This is a very complex physiological phenomenon with multiple factors to consider, some of which are modifiable. Maternal excess adiposity preconception is a modifiable correlate of low vitamin D status in the neonate [5, 6]. This is potentially due to vitamin D sequestration in maternal adipose tissue, volumetric dilution [7] and consequently hindered placental transfer of vitamin D [5]. Lower cord 25-hydroxyvitamin D [25(OH)D] concentrations are observed in newborns of mothers with BMI over 30 kg/m^2^ compared to those born to mothers with BMI in the recommended range (18.5 to 24.9 kg/m^2^), even though the mothers, on average, had sufficient vitamin D status (25(OH)D ≥ 50 nmol/L) in the third trimester [5]. In addition, neonates born to mothers with pre-gravid BMI over 25 kg/m^2^ [8] and those with gestational weight gain above the Institute of Medicine (IOM) guidelines [9] have elevated neonatal whole body fat mass and percentage body fat [10].

In Canada, 10-15% of women do not have sufficient vitamin D status [11–13]. Based on pregnancy cohort studies, inadequate maternal 25(OH)D during gestation associates with higher abdominal adiposity in neonates [14], as well as higher body fat (%) in children at 5 to 9.5 years of age [15, 16]. Similarly, mothers in the highest quartile of vitamin D status in the third trimester had children with greater lean mass (%) at 4 years of age compared to those of mothers in the lowest quartile [17]. These patterns prevailed even after adjusting for sociodemographic factors as well as maternal BMI before [14, 17] or during [15, 16] pregnancy.

Recent evidence suggests that fetal exposure to both low maternal vitamin D status and excess adiposity is associated with body composition of the offspring. The majority of these studies used BMI as a proxy measure of adiposity. Studies reporting upon lean and fat mass partitioning in both mother and neonate are scarce. The aim of the current study was to explore the correlates of maternal and neonatal vitamin D status and to assess whether the association between maternal vitamin D status and neonatal adiposity is modified by maternal adiposity preconception.

## Materials and methods

### Study design and population

Participants included mother-infant pairs (*n* = 142) who were recruited at the Lakeshore General Hospital, located in greater Montréal, Québec, Canada as part of a trial of vitamin D supplementation in breastfed infants, from March 2016 through to March 2019. The present study includes data collected prior to hospital discharge as previously published [18], and data from the baseline visit (2-6 weeks postpartum) before entering a trial (NCT02563015). The inclusion criteria for this analysis were healthy, singleton, term born infants of appropriate weight for gestational age (AGA) [19]. Exclusion criteria were: infants born to mothers with gestational diabetes or hypertension in the present pregnancy, comorbidities (liver, renal, celiac and Crohn’s diseases), medications that are known to impact vitamin D metabolism or limit growth, as well as smoking or illicit drugs [20–22].

### Obstetric history, demographic and lifestyle surveys

Prior to hospital discharge, the obstetric history including pre-pregnancy weight, weight at delivery, parity, and mode of delivery were obtained from the medical record. The mother’s pre-pregnancy BMI was then calculated using mothers’ weight prior to pregnancy and measured height at the postpartum visit. Demographic information surveyed included: maternal age and self-reported population group (white/all other groups combined including if unknown) which was defined according to the proposed guideline from Canadian Institute for Health Information [23], and in doing so any mixed ancestry were categorized as other groups. The highest level of education completed (elementary/high school, college/vocational school, or university), and household income were surveyed according to the median annual income for Canadian families with children and collapsed into ≥70,000, < 70,000 Canadian dollars (CAD), or not reported [24]. Lifestyle factors were surveyed as reported previously [18] including maternal multivitamin supplement use (yes/no) and the frequency (every day, almost every day, 2-3/week or less); and exercise habits (yes/no), typical frequency (none, 1-2 h/wk., ≥ 3 h/wk), and intensity (low, moderate or high), during the 3 months prior to pregnancy and then separately for across pregnancy. Moreover, whether the infants had received vitamin D supplements containing 400 IU/d prescribed by their physician prior to hospital discharge was surveyed at the follow-up visit.

### Biochemistry measurements

Capillary blood was sampled from the neonates between 24 and 36 h of life [18]. Mothers and their infants participated in a postnatal visit at 1 month (± 0.5 month) at the Mary Emily Clinical Nutrition Research Unit, McGill University. In the non-fasted state, capillary blood samples (0.4-0.5 ml) were collected from infants by heel lance; and a maternal venous sample (5 ml) was taken to assess vitamin D status which does not vary significantly between delivery and 1 mo postpartum [25, 26]. Total serum 25(OH)D using an automated chemiluminescence immunoassay (Liaison, DiaSorin Inc.). The laboratory maintained a certificate of proficiency from the Vitamin D External Quality Assessment Scheme. Vitamin D control samples from the National Institute of Standards and Technology (NIST) quality assurance program were implemented in routine quality control measures. The inter-assay coefficient of variation for NIST972a (levels 1 to 4) was on average < 10% and the accuracy 97.4% of certified values. The inter-assay coefficient of variation for an internal laboratory control (62.8 nmol/L) human serum sample was 8.2% across all assays. Deming regression was used to standardize the original measured 25(OH)D values for mothers and infants to NIST reference measurements: standardized concentration = 0.9634 (Liaison concentration + 3.122 nmol/L). In a subgroup of mothers and infants (*n* = 83), total 25(OH)D was in agreement (mean difference = − 0.8) with liquid chromatography tandem mass spectroscopy (Queen’s University, Kingston, Ontario, Canada) using an assay certified by the Vitamin D Standardization-Certification Program. In the current study, the cut-point for sufficiency of vitamin D status in mother-infant dyads was set at ≥50 nmol/L of serum 25(OH)D in accordance with the IOM [27] and since vitamin D status above this cut-point also positively relates to lean mass at 3-4 y [17, 28]. For ease of readability, individuals with serum 25(OH)D concentration < 50 nmol/L, were termed as insufficient to reflect the increasing risk of being insufficient as serum 25(OH)D falls below 50 nmol/L. The population cut-point of 40 nmol/L of serum 25(OH)D was not used in the present analysis since the assessment was at the level of individual mother-infant dyads.

### Skin pigmentation and UVB exposure

Skin tone of the infant was measured at the research facility by taking the average of three measurements at the inner upper arm for constitutive pigmentation (basal color) using a spectrophotometer (CM-700d/600d, Konica Minolta, USA). Individual typological angle (ITA^o^) was calculated with the L* and b* values using published equations [29]. Infants were classified into two skin tone groups (F I-III; F IV-VI) based on Fitzpatrick scales [30, 31]. Based on the strength of solar UVB, vitamin D synthesizing/vitamin D non-synthesizing periods (April 1st-October 31st/November 1st-March 31st) [32] and season (winter, spring, summer, fall) [18] at birth were used as proxies for potential vitamin D synthesis.

### Anthropometric measurements

At the research facility, infant weight, length and head circumference were measured using standard methodology [33]. Weight, length, and BMI for age z-scores were calculated using WHO growth standards and software (WHO AnthroPlus, Switzerland). Measured maternal weight and height were used to calculate BMI (kg/m^2^) [33]. Total weight gain in pregnancy was estimated by subtracting pre-pregnancy weight from the weight obtained at delivery [34] and classified as inadequate, adequate, or excess weight gain according to pre-pregnancy BMI [9].

### Body composition measurements

Body composition of infants (infant whole body software) and mothers (whole body software) was assessed in a three-compartment model using a fan-beam dual-energy X-ray absorptiometer (DXA; APEX version 13.3:3, Hologic 4500A Discovery Series, Bedford, MA) as reported in detail [33]. For quality control and quality assurance purposes, a spine phantom (Hologic phantom; No. 14774) was used at each study visit and the coefficient of variation for bone mineral content, bone mineral density, and bone area were < 1%; the radiographic uniformity tests were within established limits across the study. Whole body scans provided lean mass (g) excluding bone mineral content, fat mass (g and %); from these values, lean mass index (LMI, lean body mass (kg)/stature (m)^2^), and fat mass index (FMI, fat mass (kg)/stature (m)^2^) were then calculated using standing height for mothers and crown heel length for infants.

### Power analysis and sample size estimation

This was a convenience sample of 142 mother-infant dyads in a cross-sectional analysis at birth and 1 month postpartum as part of a randomized trial [35], and thus a retrospective power was calculated based on changes in the primary outcome (fat mass) between mothers with pre-pregnancy ≥25 kg/m^2^ and 25(OH)D < 50 (*n* = 21) and those with pre-pregnancy ≥25 kg/m^2^ and 25(OH)D ≥ 50 (*n* = 28). Power was estimated to be 75% using the procedure described by Kononoff [36] for specific data sets accounting for the fixed effects of gestational weight gain, neonatal sex, gestational age, UVB period at birth, actual age of infant at the postnatal visit, and infant length in linear fixed effects design [37].

### Statistical analysis

Data analyses were conducted using Statistical Analysis System (SAS; version 9.4, SAS Institute Inc., Cary, NC). Descriptive characteristics for mothers and infants were expressed as mean (95% confidence interval) or n (%). Mothers were classified into 1 of 4 groups according to serum 25(OH)D concentrations (insufficient: 25(OH)D < 50, sufficient: 25(OH)D ≥ 50 nmol/L) and pre-pregnancy BMI (recommended BMI: < 25, overweight/obese: ≥25 kg/m^2^). The 2 × 2 design of maternal vitamin D status and pre-pregnancy BMI formed 4 groups of interest, I-RW: insufficient-recommended weight (25(OH)D < 50 and BMI < 25 kg/m^2^), I-OW/O: insufficient-overweight/obese (25(OH)D < 50 and BMI ≥25 kg/m^2^), S-RW: sufficient-recommended weight (25(OH)D ≥ 50, BMI: < 25 kg/m^2^), and S-OW/O: sufficient-overweight/obese (25(OH)D ≥ 50 nmol/L, BMI ≥25 kg/m^2^). For maternal and neonatal characteristics at delivery and 1 mo postpartum, data were compared among these groups using a linear fixed effects model, fit using SAS PROC MIXED [37] for continuous variables, followed by post hoc Tukey’s tests with Tukey-Kramer adjustment for multiple comparisons. Chi-square or Fisher’s exact tests (frequency analysis) were used to test for differences in proportions. According to the product of the interaction effect of maternal vitamin D status and pre-pregnancy BMI, the 4 groups of interests (I-RW, I-OW/O, S-RW, and S-OW/O), frequency analysis creates 3-way crosstabulation tables. Each of the categorical variables was used in the model to stratify the crosstabulation tables followed by the the interaction term (maternal 25(OH)D*pre-pregnancy BMI) creating two 2-way tables of 25(OH)D and pre-pregnancy BMI, for each level of the categorical variable.

The interrelationship among maternal pre-pregnancy BMI, maternal 25(OH)D, and the interaction effect of these two variables with neonatal body composition (lean and fat mass, related percentages, and indices) was tested using a linear fixed effects model, fit using SAS PROC MIXED [37], and post hoc Tukey’s tests adjusted for multiple comparisons using Tukey-Kramer adjustment. Disjunctive cause criterion [38] was used for selection of covariates in the model and in accordance with factors known to be associated with body composition or vitamin D status. Fixed effects included in the model were gestational weight gain [10], neonatal sex [35], gestational age (GA) [39], UVB period at birth [32], actual age of the infants at the postnatal visit [39], and infant length [1]. Other variables that were considered in these analyses were: maternal age, family income, self-reported population group, education, multivitamin supplement use, physical activity during pregnancy as well as changes in neonatal serum 25(OH)D concentration during the postpartum period (delta 25(OH)D). These variables did not improve the model as judged by Bayesian information criterion (BIC), thus were removed from the final model. We further performed sex-stratified analysis to determine whether the associations between maternal pre-pregnancy BMI, maternal vitamin D status and neonatal body composition differ by sex. In this analysis, the three-way interaction of maternal pre-pregnancy BMI, maternal vitamin D status, and sex which was otherwise tested using the same model, appeared non-significant and was removed. The two way interaction term (maternal pre-pregnancy BMI*maternal vitamin D status) and sex were retained in the final model.

In order to determine correlates of serum 25(OH)D concentrations in mother-infant dyads, maternal and neonatal serum 25(OH)D were separately modelled (using a linear fixed effects model fit using SAS PROC MIXED) against maternal pre-pregnancy BMI (healthy BMI: < 25, overweight/obese: ≥25 kg/m^2^), postpartum BMI (healthy:18.5-24.9, overweight 25.0-29.9, obese ≥30 kg/m^2^), postpartum FMI [40] (low to normal: 4-9, excess fat: > 9-13, obese: > 13 kg/m^2^), gestational weight gain [9] (inadequate, adequate, and excess), and other important lifestyle or demographic factors including exercise before and during pregnancy (yes/no) as two separate variables, multivitamin supplement use prior to/during pregnancy (yes/no), UVB period at birth/at delivery (vitamin D synthesizing, non-synthesizing period), parity (primiparous, multiparous), maternal education (elementary/high school, college/vocational school, university), and income (≥70,000, < 70,000 CAD, or not reported). Postpartum FMI [38] was substituted in place of BMI, and differences among categories otherwise tested using the same model.

In these regression models for maternal 25(OH)D, the frequency of multivitamin supplements, the frequency and intensity of the exercise prior to/during pregnancy as well as season at birth did not improve the models and were thus removed. For the regression analysis of neonatal 25(OH)D, neonatal sex (male, female) and skin tone (F I-III, F IV-VI) were additional fixed factors included in the model. Normality of continuous data were tested using Kolmogorov-Smirnov and Shapiro-Wilk tests and the residuals were normally distributed. Levene and Bartlett tests of homogeneity of variances were used to confirm the assumptions of the post-hoc testing. Correlation tests were used to identify linear relationships between continuous variables including maternal and neonatal 25(OH)D, and maternal 25(OH)D and maternal body composition (SAS PROC CORR and PROC GLM using MANOVA option). For all tests, a *P*-value of < 0.05 was used to guide interpretation of the results.

## Results

### Maternal characteristics according to vitamin D status and pre-pregnancy BMI categories

Groups categorized based on maternal 25(OH)D and pre-pregnancy BMI were not different in terms of maternal age, country of birth, self-reported population group and education (Table 1). Although the majority of mothers (92.3% overall) took a multivitamin supplement during pregnancy, the proportion was lower in I-RW compared to the other groups. No differences were observed in the dose of vitamin D taken. A higher proportion of mothers in I-OW/O had household annual income < 70,000 CAD compared to the other groups. At the postpartum visit 98.6% of mothers were breastfeeding, 2 mothers discontinued breastfeeding between recruitment at birth and the baseline visit due to milk insufficiency.

**Table 1:** Maternal characteristics according to maternal pre-pregnancy BMI and postpartum vitamin D status

Characteristic^**1**^	All	25(OH)D < 50 nmol/L		25(OH)D ≥ 50 nmol/L		***P***-value^**2**^		
		BMI < 25 kg/m^**2**^ (***n*** = 24)	BMI ≥ 25 kg/m^**2**^ (***n*** = 21)	BMI < 25 kg/m^**2**^ (***n*** = 69)	BMI ≥ 25 kg/m^**2**^ (***n*** = 28)	25(OH)D	BMI	25(OH)D*BMI
Age at delivery, y	32.2 (31.4, 32.9)	31.0 (28.9, 33.0)	32.7 (30.1, 35.2)	31.9 (31.0, 32.8)	33.5 (31.7, 35.2)	0.29	0.05	0.93
Parity, n (%)								
Primiparous	44 (31.0)	12 (50.0)	6 (28.6)	21 (30.4)	5 (17.9)	0.23	0.0009	0.28
Multiparous	98 (69.0)	12 (50.0)	15 (71.4)	48 (69.6)	23 (82.1)	< 0.0001	0.03	0.04
Pre-pregnancy BMI, kg/m^2^	24.6 (23.8, 25.3)	22.4 (21.6, 23.2)	30.0 (28.1, 31.8)	21.8 (21.4, 22.2)	29.2 (27.3, 31.0)	0.22	< 0.0001	0.85
Gestational weight gain, kg	13.6 (12.6, 14.6)	13.3 (10.2, 16.5)	12.0 (9.3, 14.6)	14.8 (13.5, 16.1)	12.0 (9.7, 14.4)	0.49	0.07	0.53
Serum 25(OH)D, nmol/L	67.4 (63.1, 71.7)	40.0 (36.2, 43.9)	40.4 (36.8, 43.9)	83.3 (78.1, 88.6)	71.8 (64.8, 78.8)	< 0.0001	0.10	0.08
Maternal birthplace, n (%)								
Canada	90 (63.4)	13 (54.2)	8 (38.1)	50 (72.5)	19 (67.9)	< 0.0001	0.0001	0.35
Elsewhere	52 (36.6)	11 (45.8)	13 (61.9)	19 (27.5)	9 (32.1)	0.58	0.27	0.11
Self-reported population group, n (%)								
White	79 (55.6)	8 (37.5)	7 (33.3)	47 (71.0)	17 (60.7)	< 0.0001	0.0002	0.16
All other groups^3^	63 (44.4)	16 (62.5)	14 (66.7)	22 (29.0)	11 (39.3)	0.80	0.20	0.32
Supplement use^4^, n (%)								
Yes	131 (92.3)	20 (83.3)	20 (95.2)	65 (94.2)	26 (92.9)	< 0.0001	0.0007	0.02
No	11 (7.7)	4 (16.7)	1 (4.8)	4 (5.8)	2 (7.1)	0.76	0.13	0.62
Vitamin D dosage, IU/d	501.5 (478.6, 524.3)	470 (402.8, 537.2)	513.2 (458.8, 567.5)	504.6 (472, 537.1)	510.9 (453.7, 568.1)	0.53	0.34	0.48
Education, n (%)								
Elementary/high school	13 (9.2)	4 (17.8)	4 (19.0)	4 (5.8)	1 (3.6)	0.41	0.41	0.28
College/vocational school	30 (21.1)	5 (13.3)	1 (4.8)	15 (21.7)	9 (32.1)	0.001	0.07	0.33
University	99 (69.7)	15 (68.9)	16 (76.2)	50 (72.5)	18 (64.3)	0.0002	0.002	0.01
Family annual income^5^, n (%)								
≥ 70,000 CAD	80 (56.3)	15 (62.5)	5 (23.8)	42 (60.9)	18 (64.3)	< 0.0001	0.0001	0.67
< 70,000 CAD	41 (28.9)	4 (16.7)	13 (61.9)	18 (26.1)	6 (21.4)	0.27	0.64	0.001
Not reported	21 (14.8)	5 (20.8)	3 (14.3)	9 (13.0)	4 (14.3)	0.28	0.13	1.00

*Abbreviations*: *25(OH)D* 25-hydroxyvitamin D, *BMI* Body mass index, *CAD* Canadian dollar

^1^Data are mean (lower and upper 95% confidence limits) or n (%)

^2^Data were compared using a linear fixed effects model for continuous variables followed by post hoc Tukey’s tests with Tukey-Kramer adjustment for multiple comparisons and Chi-square or Fisher exact tests for categorical variables (using frequency procedure to create 3-way crosstabulation tables; categorical variables were used in the model to stratify the crosstabulation tables followed by the last two variables: maternal 25(OH)D*pre-pregnancy BMI, creating two 2-way tables of 25(OH)D and pre-pregnancy BMI, for each level of the categorical variables)

^3^Other groups included: South Asian, Chinese, Black, Filipino, Latin American, Arab, Southeast Asian, West Asian, Korean, Japanese, or other

^4^Use of prenatal supplement containing vitamin D during pregnancy

^5^The median income (in Canadian dollars) for Canadian families with children

### Neonatal characteristics at birth and postpartum according to maternal vitamin D status and pre-pregnancy BMI

Newborns (59% male) were not different among groups in terms of GA, age at the postpartum visit, UVB period at birth, and anthropometric measurements (Table 2). At birth, on average, infant serum 25(OH)D concentrations were below the cut-point for sufficiency of 50 nmol/L for both maternal pre-pregnancy BMI categories of within or above the recommended range (47.8 [95%CI: 43.8, 51.9] vs. 38.1 [95%CI: 33.5, 42.7]). At birth and 1 month of age, infants of mothers in I-RW and I-OW/O had significantly lower serum 25(OH)D concentrations compared to infants born to mothers in S-RW and S-OW/O. In addition, more infants in I-OW/O were male compared to the other groups whereas a higher proportion of infants in S-RW had skin tone F I-III compared to the rest of the groups.

**Table 2:** Neonatal characteristics according to maternal pre-pregnancy BMI and postpartum vitamin D status

Characteristic^**1**^	All	25(OH)D < 50 nmol/L		25(OH)D ≥ 50 nmol/L		***P***-value^**2**^		
		BMI < 25 kg/m^**2**^ (***n*** = 24)	BMI ≥ 25 kg/m^**2**^ (***n*** = 21)	BMI < 25 kg/m^**2**^ (***n*** = 69)	BMI ≥ 25 kg/m^**2**^ (***n*** = 28)	25(OH)D	BMI	25(OH)D*BMI
**Birth**								
Gestational age, wk	39.64 (39.5, 39.8)	39.9 (39.4, 40.4)	39.7 (39.2, 40.1)	39.7 (39.4, 39.9)	39.3 (38.9, 39.8)	0.13	0.13	0.90
Sex, n (%)								
Male	83 (58.5)	13 (54.2)	14 (66.7)	40 (58.0)	16 (57.1)	0.002	0.01	0.04
Female	59 (41.5)	11 (45.8)	7 (33.3)	29 (42.0)	12 (42.9)	0.003	0.01	0.47
UVB period^3^, n (%)								
Synthesizing	83 (58.5)	14 (58.3)	9 (42.9)	43 (62.3)	17 (60.7)	< 0.0001	0.001	0.34
Non-synthesizing	59 (41.5)	10 (41.7)	12 (57.1)	26 (37.7)	11 (39.3)	0.05	0.09	0.06
Weight, kg	3.4 (3.3, 3.5)	3.4 (3.3, 3.6)	3.5 (3.3, 3.7)	3.3 (3.3, 3.4)	3.4 (3.2, 3.6)	0.12	0.44	0.69
Weight z score	0.2 (0.0, 0.3)	0.3 (−0.1, 0.7)	0.3 (− 0.1, 0.7)	0.1 (− 0.1, 0.2)	0.2 (− 0.1, 0.5)	0.20	0.68	0.54
Serum 25(OH)D, nmol/L	44.5 (41.3, 47.6)	29.3 (24.8, 33.7)	26.6 (23.0, 30.2)	54.3 (50.0, 58.6)	46.7 (40.8, 52.5)	< 0.0001	0.07	0.38
**Postnatal visit**								
Age, mo	0.7 (0.6, 0.8)	0.7 (0.6, 0.8)	0.6 (0.5, 0.8)	0.7 (0.7, 0.8)	0.7 (0.6, 0.8)	0.37	0.25	0.51
Weight, kg	4.0 (3.9, 4.1)	4.1 (3.8, 4.3)	3.9 (3.7, 4.2)	3.9 (3.8, 4.1)	4.0 (3.8, 4.2)	0.61	0.68	0.29
Weight z score	−0.1 (− 0.2, 0.0)	0.1 (−2.0, 0.5)	− 0.01 (− 0.4, 0.4)	−0.2 (− 0.4, − 0.03)	−0.02 (− 0.4, 0.3)	0.23	0.86	0.28
Length, cm	53.1 (52.7, 53.4)	53.8 (52.9, 54.8)	53.0 (52.2, 53.8)	52.9 (52.4, 53.3)	53.0 (52.1, 53.9)	0.22	0.34	0.19
Length z score	−0.01 (− 0.2, 0.1)	0.4 (− 0.1, 0.8)	0.1 (− 0.3, 0.4)	−0.2 (− 0.4, 0.1)	−0.03 (− 0.4, 0.3)	0.06	0.57	0.20
Head circumference, cm	36.4 (36.2, 36.6)	36.5 (35.9, 37.0)	36.5 (35.9, 37.0)	36.3 (36.1, 36.6)	36.7 (36.2, 37.2)	0.95	0.40	0.43
Head circumference z score	0.1 (−0.02, 0.3)	0.2 (− 0.3, 0.6)	0.3 (− 0.1, 0.7)	−0.03 (− 0.2, 0.2)	0.3 (0.01, 0.7)	0.71	0.13	0.43
Serum 25(OH)D, nmol/L	54.7 (51.9, 57.5)	45.1 (40.3, 49.9)	42.3 (34.5, 50.1)	61.9 (57.9, 65.9)	54.5 (49.3, 59.7)	< 0.0001	0.08	0.42
Skin tone^4^, n (%)								
F I-III	110 (77.5)	17 (70.8)	14 (66.7)	59 (85.5)	20 (71.4)	< 0.0001	< 0.0001	0.04
F IV-VI	32 (22.5)	7 (29.2)	7 (33.3)	10 (14.5)	8 (28.6)	0.48	0.72	0.75

*Abbreviations*: *25(OH)D* 25-hydroxyvitamin D, *F* Fitzpatrick, *UVB* Ultraviolet B

^1^Data are mean (lower and upper 95% confidence limits) or n (%)

^2^Data were compared using a linear fixed effect model for continuous variables followed by post hoc Tukey’s tests with Tukey-Kramer adjustment for multiple comparisons; and Chi-square or Fisher exact tests for categorical variables (using frequency procedure to create 3-way cross tabulation tables; categorical variables were used in the model to stratify the crosstabulation tables followed by the last two variables: maternal 25(OH)D*pre-pregnancy BMI, creating two 2-way tables of 25(OH)D and pre-pregnancy BMI, for each level of the categorical variables)

^3^Vitamin D synthesizing: April 1st-October 31st or vitamin D non-synthesizing: November 1st-March 31st

^4^Classified based on Fitzpatrick descriptions: F I-III (light) and F IV-VI (dark) [30, 31]

Overall, the majority (95.0%) of infants received daily vitamin D supplements (containing 400 IU vitamin D) between discharge from hospital and the follow-up visit. Infant mean serum 25(OH)D concentrations significantly increased during the postnatal period (birth: 44.5 [95%CI: 41.3, 47.6] vs. 1 month: 54.7 [95%CI: 51.9, 57.5] nmol/L; *P* < 0.0001). Infants born with serum 25(OH)D ≥ 50 nmol/L had significantly lower (*P* < 0.0001) mean change in serum 25(OH)D concentration (1.4, 95%CI: − 3.0, 5.8 nmol/L) compared to neonates born with 25(OH)D 30-49.9 nmol/L (12.9, 95%CI: 10.2, 15.7 nmol/L), and those deficient < 30 nmol/L (17.7, 95%CI: 12.5, 22.8 nmol/L). Infant serum 25(OH)D concentrations at birth (*r* = 0.77; *P* < 0.0001) and at 1 month (*r* = 0.59, *P* < 0.0001) were positively correlated with maternal serum 25(OH)D concentrations. These correlations remained evident after adjusting for parity, maternal multivitamin supplement use, gestational age at birth, sex, UVB period at birth, and infant skin tone.

### Maternal vitamin D status, indicators of adiposity and whole-body lean mass

**Fig. 1:**
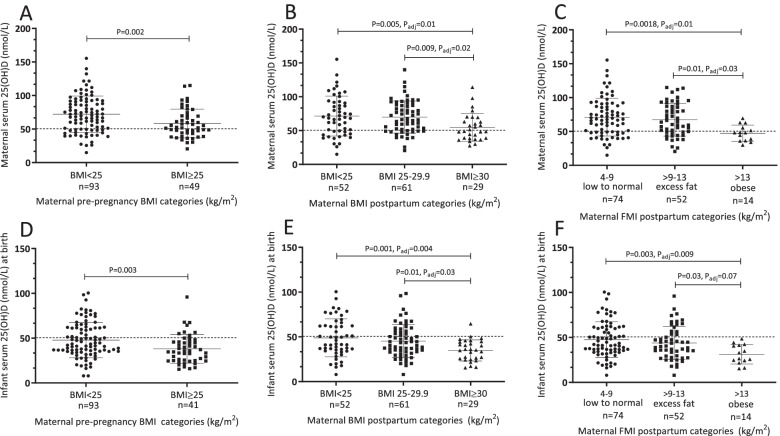
Maternal and neonatal serum 25(OH)D according to maternal BMI and FMI categories. Serum 25(OH)D concentrations of **A** mothers according to pre-pregnancy BMI categories (healthy: BMI < 25 or overweight/obese: BMI ≥25 kg/m^2^), **B** serum 25(OH)D concentrations of mothers according to postpartum BMI categories (healthy: BMI < 25, overweight: BMI 25-29.9, obese: BMI ≥30 kg/m^2^, **C** mothers according to their postpartum fat mass index (FMI) categories (low to normal: 4-9, excess fat: > 9-13, and obese: > 13 kg/m^2^). Serum 25(OH)D concentrations of infants at birth according to mothers, **D** pre-pregnancy BMI categories, **E** postnatal BMI categories and, **F** FMI categories. Data were compared using a linear fixed effects model, maternal pre-pregnancy, postpartum BMI and FMI as categorical fixed effects followed by post hoc Tukey’s tests with Tukey-Kramer adjustment for multiple comparisons. Data are mean ± SD

In mothers, serum 25(OH)D concentrations were weakly, positively correlated with whole body lean mass (*r* = 0.23, *P* = 0.006) and weakly, inversely correlated with percent whole body fat mass (*r* = − 0.26, *P* = 0.002). Maternal serum 25(OH)D was on average higher in mothers with pre-pregnancy BMI < 25 kg/m^2^ (Fig. 1A) compared to ≥25 kg/m^2^. Similarly, serum 25(OH)D was lower with postpartum BMI < 25 kg/m^2^ or 25-29.9 kg/m^2^ (Fig. 1B) compared to mothers with BMI ≥30 kg/m^2^. However, on average, maternal serum 25(OH)D concentrations were ≥ 50 nmol/L in all BMI categories (68.3 and 57.1% of mothers with BMI < 25 and ≥ 25 kg/m^2^ had 25(OH)D concentrations ≥50 nmol/L, respectively). Likewise, serum 25(OH)D of mothers with low to normal or excess FMI categories was higher compared to mothers with FMI in the obese range (Fig. 1C). Appropriateness of gestational weight gain was not related to maternal serum 25(OH)D (Supplementary Table 1). Among important correlates of vitamin D status, mothers who self-reported being physically active (indoor and outdoor combined) 3 months prior to conception or during pregnancy had higher 25(OH)D concentrations postpartum versus mothers who were not active. Additionally, self-reported population group (white/all other groups) was a prominent correlate of maternal serum 25(OH)D. Other tested covariates such as parity, multivitamin supplement use, education level and family income were not related.

### Neonatal vitamin D status and maternal indicators of adiposity

The mean serum 25(OH)D was on average higher in infants of mothers with pre-pregnancy BMI (Fig. 1D) < 25 kg/m^2^ compared to ≥25 kg/m^2^. Infant 25(OH)D was also higher in infants of mothers with postpartum BMI < 25 kg/m^2^ or 25-29.9 kg/m^2^ (Fig. 1E) compared to mothers with BMI ≥30 kg/m^2^. Similarly, serum 25(OH)D concentrations of infants born to mothers in the low to normal category of FMI were higher compared to infants born to mothers in the obese category (Fig. 1F). However, on average, infant serum 25(OH)D concentrations were below the cut-point for sufficiency of 50 nmol/L for all maternal BMI (38.7 and 20.4% of infants of mothers with < 25 and ≥ 25 kg/m^2^ had vitamin D sufficiency) or FMI categories (39.2, 30.8, and 0% of infants of mothers with FMI of low to normal: 4-9, excess fat: > 9-13, and obese: > 13 kg/m^2^, respectively). The relationship among neonatal vitamin D status and maternal indicators of adiposity and other maternal factors is shown in detail in (Supplementary Table 2).

**Fig. 2:**
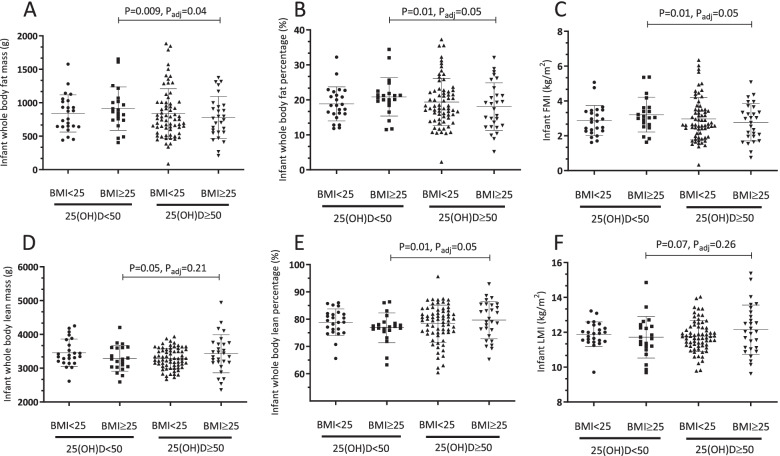
The interaction effect of maternal pre-pregnancy BMI and maternal 25(OH)D status with neonatal body composition. **A** Whole-body fat mass, **B** whole-body fat percentage, **C** fat mass index (FMI), **D** whole-body lean mass, **E** whole-body lean percentage, and **F** lean mass index (LMI). Data were compared using a linear fixed effects model, maternal pre-pregnancy BMI and 25(OH)D interaction as a categorical fixed effect followed by post hoc Tukey’s tests with Tukey-Kramer adjustment for multiple comparisons. Data are mean ± SD

Maternal pre-pregnancy BMI (< 25 kg/m^2^ or ≥ 25 kg/m^2^) and maternal serum 25(OH)D (< 50 or ≥ 50 nmol/L) as independent categorical variables were not associated with any of neonatal indicators of adiposity (Table 3 and Supplementary Table 3). However, a significant interaction effect of maternal pre-pregnancy BMI and 25(OH)D concentration was observed for neonatal fat mass (Table 3), fat percentage and FMI adjusting for multiple covariates (Supplementary Table 3). After pairwise comparison tests, infants of mothers in the I-OW/O group with elevated pre-pregnancy BMI (≥25 kg/m^2^) and vitamin D insufficiency (25(OH)D < 50 nmol/L) had significantly higher fat mass (Fig. 2A), fat percentage (Fig. 2B), and FMI (Fig. 2C) compared to infants born to mothers in the S-OW/O group with BMI > 25 kg/m^2^ but vitamin D sufficiency (25(OH)D ≥ 50 nmol/L). The magnitude of difference in whole body fat mass observed between I-OW/O and S-OW/O groups was 220.4 g (95% CI: 56.4, 384.3) representing ~ 28% difference with effect size of 0.42. Whole body fat mass, fat percentage, and FMI were also higher in female infants versus males (*P* < 0.05) (Supplemental Fig. 1A, B, C) and in infants born in the vitamin D synthesizing period (UVB period) versus those born vitamin D non-synthesizing period (*P* < 0.05) (Supplemental Table 3). Other covariates linked to greater infant adiposity indicators were infant age and length (Table 3 and Supplemental Table 3). In these adjusted mixed models, gestational weight gain was not associated with neonatal fat mass or any other adiposity indicators.

**Table 3:** Correlates of neonatal body composition

Fixed effects model^**a**^	Regression coefficients	95% Confidence intervals	***P***-value	Adjusted ***P***-value
**Neonatal whole-body fat mass g (*R***^**2**^ **0.38,** ***R***^**2**^_**adj**_ **0.37)**^a^				
Sex^b^ of infant (Ref: female)	− 119.85	− 219.24, − 20.45	**0.02**	
Gestational age at birth, wk	−15.38	−67.13, 36.38	0.56	
Infant age, mo	435.35	187.62, 683.09	**0.001**	
Infant length, cm	58.80	29.99, 87.57	**< 0.0001**	
UVB period at birth^c^ (Ref: non-synthesizing period)	105.28	8.94, 201.63	**0.03**	
Gestational weight gain, kg	6.57	−1.61, 14.76	0.11	
Maternal pre-pregnancy BMI^d^ (Ref: < 25 kg/m^2^)	− 45.26	− 173.54, 83.02	0.12	
Maternal 25(OH)D^e^ (Ref: ≥50 nmol/L)	− 40.57	− 176.04, 94.91	0.09	
BMI*25(OH)D interaction (pairwise comparisons)			**0.02**	
BMI ≥ 25, 25(OH)D < 50 vs BMI ≥ 25, 25(OH)D ≥ 50	220.40	56.19, 384.60	**0.009**	**0.04**
BMI ≥ 25, 25(OH)D < 50 vs BMI < 25, 25(OH)D < 50	215.70	44.73, 386.68	**0.01**	0.07
BMI ≥ 25, 25(OH)D < 50 vs BMI < 25, 25(OH)D ≥ 50	175.14	28.38, 321.89	**0.02**	0.09
BMI ≥ 25, 25(OH)D ≥ 50 vs BMI < 25, 25(OH)D < 50	−4.70	− 161.54, 152.15	0.95	0.99
BMI ≥ 25, 25(OH)D ≥ 50 vs BMI < 25, 25(OH)D ≥ 50	−45.26	−173.54, 83.02	0.49	0.90
BMI < 25, 25(OH)D < 50 vs BMI < 25, 25(OH)D ≥ 50	−40.57	−176.04, 94.91	0.55	0.93
**Neonatal whole-body lean mass g (*R***^**2**^ **0.53,** ***R***^**2**^_**adj**_ **0.52)**				
Sex of infant (Ref: female)	174.52	73.41, 275.64	**0.0009**	
Gestational age at birth, wk	32.29	−20.36, 84.94	0.23	
Infant age, mo	294.98	42.95, 547.00	**0.02**	
Infant length, cm	102.26	72.97, 131.55	**< 0.0001**	
UVB period (Ref: non-synthesizing period)	−68.86	− 166.88, 29.16	0.17	
Gestational weight gain, kg	−4.56	−12.89, 3.76	0.28	
Maternal pre-pregnancy BMI (Ref: < 25 kg/m^2^)	124.67	−5.83, 255.17	0.06	
Maternal 25(OH)D (Ref: ≥50 nmol/L)	55.67	−82.15, 193.49	0.43	
Maternal pre-pregnancy BMI*25(OH)D			**0.04**	
BMI ≥ 25, 25(OH)D < 50 vs BMI ≥ 25, 25(OH)D ≥ 50	− 164.58	−384.34, 55.19	**0.05**	0.21
BMI ≥ 25, 25(OH)D < 50 vs BMI < 25, 25(OH)D < 50	−95.57	− 324.39, 133.24	0.28	0.70
BMI ≥ 25, 25(OH)D < 50 vs BMI < 25, 25(OH)D ≥ 50	−39.91	− 236.31, 156.50	0.60	0.95
BMI ≥ 25, 25(OH)D ≥ 50 vs BMI < 25, 25(OH)D < 50	69.00	− 140.90, 278.91	0.39	0.83
BMI ≥ 25, 25(OH)D ≥ 50 vs BMI < 25, 25(OH)D ≥ 50	124.67	−47.01, 296.35	0.06	0.24
BMI < 25, 25(OH)D < 50 vs BMI < 25, 25(OH)D ≥ 50	55.67	− 125.64, 236.97	0.43	0.85

*Abbreviations*: *25(OH)D* 25-hydroxyvitamin D, *BMI* Body mass index, *UVB* Ultraviolet B

^a^Data were compared using a linear fixed effect model for continuous variables followed by post hoc Tukey’s tests with Tukey-Kramer adjustment for multiple comparisons

^b^Sex of infant (male vs. female)

^c^UVB period (April 1st-October 31st or November 1st-March 31st)

^d^Maternal pre-pregnancy BMI (BMI < 25 kg/m^2^ or BMI ≥25 kg/m^2^)

^e^Maternal serum 25(OH)D (≥ or < 50 nmol/L)

Similar to neonatal indicators of adiposity, maternal pre-pregnancy BMI (< 25 kg/m^2^ or ≥ 25 kg/m^2^) and maternal serum 25(OH)D (< 50 or ≥ 50 nmol/L) were not independently linked to lean mass, lean percentage and LMI in the infants (Table 3 and Supplementary Table 4). A significant interaction effect of maternal pre-pregnancy BMI and 25(OH)D concentration was observed for neonatal lean mass (Table 3 and Fig. 2D) and lean percentage (Fig. 2E) but not LMI (Fig. 2F). However, after pairwise comparison tests, only infants of mothers in I-OW/O with BMI ≥25 kg/m^2^ before pregnancy and vitamin D insufficiency (25(OH)D < 50 nmol/L) had significantly lower lean percentage compared to infants born to mothers in S-OW/O with BMI ≥25 kg/m^2^ but with vitamin D sufficiency 25(OH)D ≥ 50 nmol/L (*P* = 0.05) (Supplementary Table 4). Whole body lean mass, percentage lean mass, and LMI were also observed to be higher in male infants versus females (*P* < 0.05) (Supplemental Fig. 1D, E, F). In addition, only percentage lean mass appeared to be lower in infants born in the vitamin D synthesizing period (UVB period) versus those born in vitamin D non-synthesizing period (*P* = 0.0482) (Supplemental Table 4). Infant age and length were important correlates of lean mass and percentage lean mass, (Table 3 and Supplemental Table 3).

## Discussion

According to our data, healthy neonates dually exposed to insufficient maternal vitamin D status and elevated pre-pregnancy BMI had higher whole body fat mass (∆ 220.4 g, ~ 28% difference) compared to those of mothers with elevated pre-pregnancy BMI yet vitamin D sufficient. Our results are unique in examining maternal adiposity and vitamin D status as dual exposures in programming of neonatal body composition and complement other reports that separately link maternal vitamin D status to adiposity in the neonatal period [14] or in childhood [15, 16], and maternal overweight/obese pre-gravid BMI with neonatal adiposity [8, 41].

Most national guidelines for a healthy pregnancy [42, 43] do not have a specific recommendation on vitamin D supplementation for pregnant women with an elevated BMI; nor their newborn. Although the majority of mothers took multivitamin supplements containing vitamin D during pregnancy, 64.3% of infants of mothers with elevated pre-pregnancy BMI had serum 25(OH)D < 50 nmol/L. If maternal serum 25(OH)D was < 50 nmol/L all infants had 25(OH)D < 50 nmol/L and the majority (71.4%) were vitamin D deficient. These observations reinforce the importance of encouraging overweight/obese women to seek nutrition counselling prior to conception [44] or if pregnant to initiate the consultation and multivitamin supplementation as soon as possible to help establish vitamin D stores in the fetus and ultimately in the newborn [5]. This should be followed by neonatal vitamin D supplementation [27].

This study adds that maternal 25(OH)D concentration is an influential factor in neonatal vitamin D status at birth and within the neonatal period. We observed a positive correlation between maternal and neonatal vitamin D status at birth, which decreases 23.4% through the neonatal period, in line with other reports [45, 46]. This is attributed to the fact that the fetus is fully reliant on maternal-fetal transfer of vitamin D [47], however, shortly after birth the majority of infants commenced routine supplements containing 400 IU/d vitamin D and were breastfed. Overall, the majority of infants at birth (67.6%) had serum 25(OH)D < 50 nmol/L, whereas at the postpartum visit, this declined to 40%. Even though 4 ~ 6 week is not long enough to see a plateau in the response to vitamin D supplementation [48–50], the increments in vitamin D status of infants with 25(OH)D < 50 nmol/L agree with other reports [51, 52]. The response of infants to vitamin D supplementation inversely related to basal status resulting in a greater increment in serum 25(OH)D in neonates with vitamin D deficiency. Thus, 400 IU/d of vitamin D is suitable for healthy term-born infants, even if born with vitamin D deficiency.

Vitamin D status of mothers could be a proxy for other healthy behaviors, reflect quality of diet, or time spent outside. In our study exercise (combined indoor and outdoor) in pregnancy was associated with 14.8 nmol/L higher 25(OH)D concentration in mothers of all BMI ranges. This is of relevance since exercise tends to take place outdoors [53] with a positive association between exercise and time spent outside [54, 55]. Outdoor activity and being more exposed to sunlight promotes vitamin D synthesis and combined with vitamin D mobilization from adipose tissue [56] supports achievement and maintenance of higher vitamin D status. Physical activity is encouraged as part of a healthy lifestyle in pregnancies without contraindications [57].

The genes that regulate fat distribution, adiposity [58], and skeletal muscle phenotypes [59] are responsive to environmental and lifestyle exposures. Achieving appropriate body weight and being physically active preconception and during pregnancy may determine body composition in the offspring [60]. Regular physical activity shifts the increased energy demands to maternal muscle mass and away from the adipocytes of the fetus leading to proportionately increased lean mass and decreased adipose tissue [60]. This pattern is consistent with the observations in our study and is likely due to mobilization of vitamin D from fat tissue into circulation [56]. Therefore, it can be inferred that genetic, behavioral and environmental interactions contribute to variations in fat-muscle partitioning in early development.

In this study, we did not observe an association between maternal vitamin D status and infant lean body mass, potentially due to all infants being born term and AGA. In comparison to infants born AGA, body composition partitioning differs among infants at the extremes of birth weight, small or large for gestational age [61]. Infants born large for gestational age tend to have vitamin D insufficiency [62, 63], possibly due to entrapment of vitamin D in fat tissue compared to AGA. In addition, by including only non-smoking mothers we eliminated smoking as a confounder as neonates born to smoking mothers have lower vitamin D status [64] as well as lower lean body mass [65, 66]. Lack of association between vitamin D and infant lean body mass might also reflect the rapid growth spurt in the first month of life. Furthermore, sex differences in infant body composition emerged early postnatally. This effect was independent of the UVB period at birth, GA, maternal supplement use, and maternal and neonatal vitamin D status. Male infants have more muscle mass due to the anabolic effect of testosterone which temporarily surges postnatally within 1 ~ 3 months postpartum [67, 68]. In contrast, in our study and others, females have greater stores of fat mass irrespective of vitamin D status [69].

### Strengths, limitations, and further research

Strengths of this study are inclusion of infants of a diverse ancestry which aids the generalizability of the findings to multi-ethnic populations and assessment of body composition early in postnatal period in mother infant dyads. There are however some limitations including that pre-pregnancy weight was obtained from medical records, some of which could be self-reported. Pre-pregnancy BMI as a proxy for overweight and obesity may underestimate or misclassified adiposity [70], however, the three-compartmental model of DXA confirmed excess adipose tissue. We used an immunoassay to measure total serum 25(OH)D which is not a gold standard technique, nonetheless, the manufacturer (DiaSorin) is certified by the Vitamin D Standardization-Certification Program [71]. Furthermore, we used rigorous quality assurance measures and standardized 25(OH)D concentrations to NIST standard reference materials. Given the design of the study, maternal 25(OH)D concentrations were only measured at the postpartum visit and not at delivery. This may not be a major limitation as maternal 25(OH)D concentrations are not significantly different between 36 weeks of GA and 1 month postpartum [25, 26]. Additionally, our analysis might have been statistically underpowered to detect relationships such as the association between gestational weight gain and maternal or neonatal vitamin D status, future larger studies are needed. Lastly, whether the body composition pattern extends later into childhood requires a longitudinal study.

## Conclusion

In otherwise healthy mother-infant dyads, maternal overweight/obesity and serum 25(OH)D < 50 nmol/L are dual exposures that associate with neonatal serum 25(OH)D < 50 nmol/L as well as higher adiposity. More concerning, 71.4% of neonates in this cohort were vitamin D deficient. These results reinforce the importance of postnatal vitamin D supplementation in infants born to mothers with BMI ≥25 kg/m^2^. In the event of low maternal-fetal transfer of vitamin D, postnatal supplementation with 400 IU/d of vitamin D readily builds vitamin D stores and in doing so may limit the impact of fetal exposures.

## Supplementary Information

**Additional file 1: Supplemental Table 1.** Maternal serum 25(OH)D postpartum based on maternal characteristics. **Supplemental Table 2.** Neonatal serum 25(OH)D at birth based on maternal characteristics. **Supplemental Table 3.** Correlates of neonatal body fat mass. **Supplemental Table 4.** Correlates of neonatal body lean mass. **Supplemental Figure 1.** Sex dimorphism in neonatal body composition.

## Data Availability

The dataset used and/or analysed during the current study will not be made publicly available because permission to share data was not requested at the time of obtaining participant consent but are available from the corresponding author on reasonable request.

## Abbreviations

25(OH)D: 25-hydroxyvitamin D
AGA: Appropriate for gestational age
BMI: Body mass index
UVB: Ultraviolet B
