# Supplementary material for: Maternal excess adiposity and serum 25-hydroxyvitamin D < 50 nmol/L are associated with elevated whole body fat mass in healthy breastfed neonates
Source: BMC Pregnancy Childbirth. 2022 Jan 29;22:83. doi: 10.1186/s12884-022-04403-w (PMC8801116; doi:10.1186/s12884-022-04403-w)
Supplement: Supplementary file 1 — Additional file 1: Supplemental Table 1. Maternal serum 25(OH)D postpartum based on maternal characteristics. Supplemental Table 2. Neonatal serum 25(OH)D at birth based on maternal characteristics. Supplemental Table 3. Correlates of neonatal body fat mass. Supplemental Table 4. Correlates of neonatal body lean mass. Supplemental Figure 1. Sex dimorphism in neonatal body composition. [file 12884_2022_4403_MOESM1_ESM.docx]

| **Maternal excess adiposity and serum 25-hydroxyvitamin D <50 nmol/L are associated with elevated whole body fat mass in healthy breastfed neonates**  Maryam Razaghi, Nathalie Gharibeh, Catherine A. Vanstone, Olusola F. Sotunde,  Shu Qin Wei, Dayre McNally, Frank Rauch, Glenville Jones, Hope A. Weiler  **Supplementary Tables:**  **Supplemental Table 1** Maternal serum 25(OH)D postpartum based on maternal characteristics | | | |
| --- | --- | --- | --- |
| **Explanatory variables**^1^ | ***n*** | **Maternal serum 25(OH)D**^2^ | **Adjusted P-value**^3^ |
| Pre-pregnancy BMI, kg/m^2^  18.5-24.9  25.0-29.9  ≥30 | 93  34  15 | 72.2^a^ (66.6, 77.8)  61.1^ab^ (53.1, 69.0)  50.0^bc^ (41.6, 58.3) | 0.003 |
| Postpartum BMI, kg/m^2^  18.5-24.9  25.0-29.9  ≥30 | 52  61  29 | 71.4^a^ (63.2, 79.6)  70.0^a^ (63.9, 76.1)  53.9^b^ (46.1, 61.6) | 0.007 |
| Postpartum FMI, kg/m^2^  Low to normal: 4-9  Excess fat: <9-13  Obese >13 | 74  52  15 | 71.1^a^ (64.6, 77.5)  67.5^a^ (60.7, 74.3)  47.5^b^ (40.6, 54.4) | 0.02 |
| Gestational weight gain, kg  Inadequate  Adequate  Excess | 35  47  59 | 63.0 (54.8, 71.1)  71.9 (64.1, 79.7)  66.1 (59.1, 73.1) | 0.41 |
| Exercise prior conceiving  Yes  No | 95  47 | 72.3 (67.1, 77.4)  56.9 (49.7, 64.2) | 0.03 |
| Exercise during pregnancy  Yes  No | 70  72 | 74.9 (68.5, 81.3)  59.7 (54.2, 65.1) | 0.007 |
| Multivitamin use prior to conceiving  Yes  No | 65  77 | 71.4 (65.2, 77.6)  63.6 (57.6, 69.6) | 0.72 |
| Multivitamin use during pregnancy  Yes  No | 131  11 | 67.7 (63.3, 72.3)  59.2 (40.5, 77.9) | 0.20 |
| Canadian-born  Yes  No | 90  52 | 72.2 (67.4, 78.0)  58.2 (51.2, 65.2) | 0.006 |
| Self-reported population group  White  All other groups | 82  60 | 74.2 (68.7, 79.6)  58.1 (51.7, 64.6) | 0.0002 |
| UVB period at delivery  Vitamin D synthesizing (Apr-Oct)  Vitamin D non-synthesizing (Nov-Mar) | 83  59 | 70.1 (64.7, 75.5)  63.0 (55.9, 70.1) | 0.11 |
| Season at delivery |  |  | 0.32 |
| Spring | 41 | 69.3 (61.6, 77.1) |  |
| Summer | 32 | 72.3 (62.9, 81.7) |  |
| Fall | 31 | 66.6 (58.4 ,74.8) |  |
| Winter | 38 | 61.9 (51.9, 71.8) |  |
| Parity  Primiparous (1)  Multiparous (≥2) | 44  98 | 62.3 (54.5, 70.0)  69.3 (64.1, 74.6) | 0.17 |
| Education  Elementary/High school  College/vocational school  University degree | 13  30  99 | 55.2 (40.3, 70.1)  71.5 (60.4, 82.6)  67.8 (62.8, 72.7) | 0.16 |
| Family income (CAD)  ≥70,000  <70,000  Refused to answer | 80  41  21 | 71.3 (65.3, 77.3)  61.3 (54.1, 68.5)  62.9 (50.6, 75.2) | 0.35 |
| ^1^Linear fixed effects model, additional fixed effects: maternal age; ^2^data are mean (lower and upper 95% confidence limits); ^3^adjusted for Tukey-Kramer post-hoc testing. The means with different superscript letters a, b, and c are significantly different. Abbreviations: 25(OH)D: 25-hydroxyvitamin D; BMI: body mass index; CAD: Canadian dollar; F: Fitzpatrick; FMI: fat mass index; UVB: ultraviolet B. | | | |

| **Supplemental Table 2** Neonatal serum 25(OH)D at birth based on maternal characteristics | | | |
| --- | --- | --- | --- |
| **Explanatory variables**^1^ | ***n*** | **Neonatal serum 25(OH)D**^2^ | **Adjusted P-value**^3^ |
| Maternal 25(OH)D, nmol/L  ≥50  <50 | 97  45 | 52.1 (48.6, 55.6)  28.0 (25.2, 30.7) | <0.0001 |
| Maternal pre-pregnancy BMI, kg/m^2^  18.5-24.9  25.0-29.9  ≥30 | 93  34  15 | 47.8^a^ (43.8, 51.9)  40.1^ab^ (34.5, 48.9)  31.2^bc^ (25.0, 37.4) | 0.004 |
| Maternal postpartum FMI, kg/m^2^  Low to normal: 4-9  Excess fat: <9-13  Obese: >13 | 74  52  14 | 47.5^a^ (43.0, 52.1)  43.8^a^ (38.7, 50.0)  31.2^b^ (30.2, 38.9) | 0.02 |
| Gestational weight gain, kg  Inadequate  Adequate  Excess | 35  47  59 | 40.6 (34.4, 46.8)  48.3 (42.4, 54.1)  43.7 (38.8, 48.5) | 0.47 |
| Infant sex  Male  Female | 83  59 | 43.6 (39.6, 47.6)  45.7 (40.5, 50.9) | 0.77 |
| Skin tone  F I-III  F IV-VI | 110  32 | 46.9 (43.3, 50.6)  36.0 (30.2, 41.8) | 0.06 |
| UVB period at birth  Synthesizing period (Apr-Oct)  Non-synthesizing period (Nov-Mar) | 83  59 | 46.9 (42.8, 51.0)  41.1 (36.2, 45.9) | 0.02 |
| Season at birth |  |  | 0.04 |
| Spring | 41 | 44.4^ab^ (39.5, 49.3) |  |
| Summer | 32 | 50.6^a^ (42.4, 58.8) |  |
| Fall | 31 | 44.0^ab^ (38.5, 49.5) |  |
| Winter | 38 | 39.8^bc^ (32.9, 46.7) |  |
| Maternal exercise prior conceiving  Yes  No | 95  47 | 48.1 (44.1, 52.1)  37.2 (32.6, 41.7) | 0.11 |
| Maternal exercise during pregnancy  Yes  No | 70  72 | 48.5 (44.1, 52.9)  40.5 (36.1, 44.9) | 0.03 |
| Maternal supplement use prior to conceiving  Yes  No | 65  77 | 48.7 (43.9, 53.5)  40.9 (36.8, 45.0) | 0.38 |
| Maternal supplement use during pregnancy  Yes  No | 131  11 | 44.9 (41.7, 48.1)  39.2 (22.5, 56.0) | 0.31 |
| Canadian-born  Yes  No | 90  52 | 48.7 (44.7, 52.7)  37.1 (32.4, 41.9) | 0.0003 |
| Self-reported population group  White  All other groups | 37.0 | 49.9 (45.7, 54.1)  37.0 (32.9, 41.2) | <0.0001 |
| Education  Elementary/High school  College/vocational school  University degree | 13  30  99 | 35.2 (24.8, 45.5)  45.0 (37.4, 52.6)  45.5 (41.8, 49.3) | 0.18 |
| Family income  ≥70,000 CAD  <70,000 CAD  Refused to answer | 80  41  21 | 48.0^a^ (43.6, 52.5)  38.4^b^ (33.4, 43.4)  42.7^ab^ (34.3, 51.2) | 0.03 |
| ^1^Linear fixed effects model; additional fixed effect: gestational age; ^2^data are mean (lower and upper 95% confidence limits); ^3^adjusted for Tukey post-hoc testing. The means with different superscript letters a, b, and c are significantly different. Abbreviation: 25(OH)D: 25-hydroxyvitamin D; BMI: body mass index; CAD: Canadian dollar; F: Fitzpatrick; FMI: fat mass index; UVB: ultraviolet B. | | | |

| Supplemental Table 3 Correlates of neonatal body fat mass | | | | |
| --- | --- | --- | --- | --- |
| Linear fixed effects model^1^ | **Regression coefficients** | **95% Confidence intervals** | **P-value** | **Adjusted P-value** |
| Neonatal whole-body fat mass g (R^2^ 0.38, R^2^_adj_ 0.37)^1^ | | | | |
| Sex^2^ of infant (Ref: female) | -119.85 | -219.24, -20.45 | 0.02 |  |
| Gestational age at birth, wk | -15.38 | -67.13, 36.38 | 0.55 |  |
| Infant age, mo | 435.35 | 187.62, 683.09 | 0.0007 |  |
| Infant length, cm | 58.80 | 29.99, 87.57 | 0.0001 |  |
| UVB period at birth^3^ (Ref: non-synthesizing period) | 105.28 | 8.94, 201.63 | 0.03 |  |
| Gestational weight gain, kg | 6.57 | -1.61, 14.76 | 0.11 |  |
| Maternal pre-pregnancy BMI^4^ (Ref: <25 kg/m^2^) | -45.26 | -173.54, 83.02 | 0.11 |  |
| Maternal 25(OH)D^5^ (Ref: ≥50 nmol/L) | -40.57 | -176.04, 94.91 | 0.10 |  |
| BMI*25(OH)D interaction (pairwise comparisons) | | | 0.02 |  |
| BMI≥25, 25(OH)D<50 vs BMI≥25, 25(OH)D≥50 | 220.40 | 56.19, 384.60 | 0.01 | 0.04 |
| BMI≥25, 25(OH)D<50 vs BMI<25, 25(OH)D<50 | 215.70 | 44.73, 386.68 | 0.01 | 0.06 |
| BMI≥25, 25(OH)D<50 vs BMI<25, 25(OH)D≥50 | 175.14 | 28.38, 321.89 | 0.02 | 0.09 |
| BMI≥25, 25(OH)D≥50 vs BMI<25, 25(OH)D<50 | -4.70 | -161.54, 152.15 | 0.98 | 1.00 |
| BMI≥25, 25(OH)D≥50 vs BMI<25, 25(OH)D≥50 | -45.26 | -173.54, 83.02 | 0.50 | 0.91 |
| BMI<25, 25(OH)D<50 vs BMI<25, 25(OH)D≥50 | -40.57 | -176.04, 94.91 | 0.54 | 0.93 |
| Neonatal fat percentage % (R^2^ 0.26, R^2^_adj_ 0.25) | | | | |
| Sex of infant (Ref: female) | -3.34 | -5.40, -1.36 | 0.001 |  |
| Gestational age at birth, wk | -0.37 | -1.43, 0.68 | 0.48 |  |
| Infant age, mo | 5.97 | 0.91, 11.0 | 0.02 |  |
| Infant length, cm | 0.67 | 0.08, 1.26 | 0.03 |  |
| UVB period at birth (Ref: non-synthesizing period) | 2.04 | 0.08, 4.00 | 0.04 |  |
| Gestational weight gain, kg | 0.14 | -0.03, 0.31 | 0.10 |  |
| Maternal pre-pregnancy BMI (Ref: <25 kg/m^2^) | -1.02 | -3.64, 1.60 | 0.14 |  |
| Maternal 25(OH)D (Ref: ≥50 nmol/L) | -0.89 | -3.65, 1.88 | 0.12 |  |
| BMI*25(OH)D (pairwise comparisons) | | |  | 0.02 |
| BMI≥25, 25(OH)D<50 vs BMI≥25, 25(OH)D≥50 | 4.38 | -0.03, 8.79 | 0.01 | 0.05 |
| BMI≥25, 25(OH)D<50 vs BMI<25, 25(OH)D<50 | 4.25 | -0.34, 8.83 | 0.02 | 0.07 |
| BMI≥25, 25(OH)D<50 vs BMI<25, 25(OH)D≥50 | 3.36 | -0.58, 7.30 | 0.03 | 0.12 |
| BMI≥25, 25(OH)D≥50 vs BMI<25, 25(OH)D<50 | -0.13 | -4.34, 4.08 | 0.97 | 1.00 |
| BMI≥25, 25(OH)D≥50 vs BMI<25, 25(OH)D≥50 | -0.89 | -4.46, 2.43 | 0.46 | 0.88 |
| BMI<25, 25(OH)D<50 vs BMI<25, 25(OH)D≥50 | -3.34 | -4.5, 2.75 | 0.51 | 0.91 |
| Neonatal fat mass index kg/m^2^ (R^2^ 0.53, R^2^_adj_ 0.52) |  |  |  |  |
| Sex of infant (Ref: female) | -0.23 | -0.42, -0.05 | 0.02 |  |
| Gestational age at birth, wk | -0.03 | -0.12, 0.07 | 0.58 |  |
| Infant age, mo | 1.81 | 0.34, 1.27 | 0.0008 |  |
| Infant length, cm | 0.08 | 0.03, 0.14 | 0.003 |  |
| UVB period at birth (Ref: non-synthesizing period) | 0.19 | 0.01, 0.37 | 0.04 |  |
| Gestational weight gain, kg | 0.01 | -0.003, 0.03 | 0.11 |  |
| Maternal pre-pregnancy BMI (Ref: ≥25 kg/m^2^) | -0.08 | -0.32, 0.16 | 0.11 |  |
| Maternal 25(OH)D (Ref: ≥50 nmol/L) | -0.08 | -0.33, 0.18 | 0.11 |  |
| BMI*25(OH)D (pairwise comparisons) | | |  | 0.02 |
| BMI≥25, 25(OH)D<50 vs BMI≥25, 25(OH)D≥50 | 0.40 | -0.0001, 0.81 | 0.01 | 0.05 |
| BMI≥25, 25(OH)D<50 vs BMI<25, 25(OH)D<50 | 0.40 | -0.02, 0.82 | 0.01 | 0.06 |
| BMI≥25, 25(OH)D<50 vs BMI<25, 25(OH)D≥50 | 0.32 | -0.04, 0.68 | 0.02 | 0.09 |
| BMI≥25, 25(OH)D≥50 vs BMI<25, 25(OH)D<50 | -0.002 | -0.39, 0.38 | 0.98 | 1.00 |
| BMI≥25, 25(OH)D≥50 vs BMI<25, 25(OH)D≥50 | -0.08 | -0.39, 0.23 | 0.52 | 0.92 |
| BMI<25, 25(OH)D<50 vs BMI<25, 25(OH)D≥50 | -0.08 | -0.41, 0.26 | 0.53 | 0.92 |
| ^1^Data were compared using a linear fixed effects model for continuous variables followed by post hoc Tukey’s tests with Tukey-Kramer adjustment for multiple comparisons; ^2^Sex of infant (male vs. female); ^3^UVB period (April 1^st^-October 31^st^ or November 1^st^-March 31^st^); ^4^Maternal pre-pregnancy BMI (BMI <25 kg/m^2^ or BMI ≥25 kg/m^2^); ^5^maternal serum 25(OH)D (≥ or <50 nmol/L 25(OH)D (≥ or <50 nmol/L). Abbreviations: 25(OH)D: 25-hydroxyvitamin D; BMI: body mass index; UVB: ultraviolet B. | | | | |

| Supplemental Table 4 Correlates of neonatal body lean mass | | | | |
| --- | --- | --- | --- | --- |
| Linear fixed effects model | **Regression coefficients** | **95% Confidence intervals** | **P-value** | **Adjusted P-value** |
| Neonatal whole-body lean mass g (R^2^ 0.53, R^2^_adj_ 0.52)^1^ | | | |  |
| Sex^2^ of infant (Ref: female) | 174.52 | 73.41, 275.64 | 0.0008 |  |
| Gestational age at birth, (wk) | 32.29 | -20.36, 84.94 | 0.23 |  |
| Infant age, (mo) | 294.98 | 42.95, 547.00 | 0.02 |  |
| Infant length, (cm) | 102.26 | 72.97, 131.55 | <0.0001 |  |
| UVB period^3^ (Ref: non-synthesizing period) | -68.86 | -166.88, 29.16 | 0.17 |  |
| Gestational weight gain, (kg) | -4.56 | -12.89, 3.76 | 0.31 |  |
| Maternal pre-pregnancy BMI^4^ (Ref: <25 kg/m^2^) | 124.67 | -5.83, 255.17 | 0.80 |  |
| Maternal 25(OH)D^5^ (Ref: ≥50 nmol/L) | 55.67 | -82.15, 193.49 | 0.33 |  |
| Maternal pre-pregnancy BMI*25(OH)D | | | 0.04 |  |
| BMI≥25, 25(OH)D<50 vs BMI≥25, 25(OH)D≥50 | -164.58 | -384.34, 55.19 | 0.05 | 0.21 |
| BMI≥25, 25(OH)D<50 vs BMI<25, 25(OH)D<50 | -95.57 | -324.39, 133.24 | 0.27 | 0.69 |
| BMI≥25, 25(OH)D<50 vs BMI<25, 25(OH)D≥50 | -39.91 | -236.31, 156.50 | 0.60 | 0.95 |
| BMI≥25, 25(OH)D≥50 vs BMI<25, 25(OH)D<50 | 69.00 | -140.90, 278.91 | 0.41 | 0.84 |
| BMI≥25, 25(OH)D≥50 vs BMI<25, 25(OH)D≥50 | 124.67 | -47.01, 296.35 | 0.06 | 0.24 |
| BMI<25, 25(OH)D<50 vs BMI<25, 25(OH)D≥50 | 55.67 | -125.64, 236.97 | 0.41 | 0.84 |
| Neonatal lean percentage % (R^2^ 0.03, R^2^_adj_ 0.03) | | | |  |
| Sex of infant (Ref: female) | 3.32 | 1.28, 5.35 | 0.001 |  |
| Gestational age at birth, (wk) | 0.34 | -0.72, 1.40 | 0.51 |  |
| Infant age, (mo) | -5.78 | -10.85, -0.70 | 0.03 |  |
| Infant length, (cm) | -0.65 | -1.24, -0.06 | 0.03 |  |
| UVB period (Ref: non-synthesizing period) | -2.0108 | -3.98, -0.04 | 0.05 |  |
| Gestational weight gain, (kg) | -0.15 | -0.32, 0.02 | 0.07 |  |
| Maternal pre-pregnancy BMI (Ref: <25 kg/m^2^) | 1.00 | -1.62, 3.63 | 0.14 |  |
| Maternal 25(OH)D (Ref: ≥50 nmol/L) | 0.85 | -1.92, 3.62 | 0.11 |  |
| Maternal pre-pregnancy BMI*25(OH)D | | | 0.02 |  |
| BMI≥25, 25(OH)D<50 vs BMI≥25, 25(OH)D≥50 | -4.4178 | -8.84, 0.01 | 0.01 | 0.05 |
| BMI≥25, 25(OH)D<50 vs BMI<25, 25(OH)D<50 | -4.2678 | -8.88, 0.34 | 0.02 | 0.07 |
| BMI≥25, 25(OH)D<50 vs BMI<25, 25(OH)D≥50 | -3.4141 | -7.37, 0.54 | 0.03 | 0.11 |
| BMI≥25, 25(OH)D≥50 vs BMI<25, 25(OH)D<50 | 0.1500 | -4.08, 4.38 | 0.96 | 1.00 |
| BMI≥25, 25(OH)D≥50 vs BMI<25, 25(OH)D≥50 | 1.0037 | -2.45, 4.46 | 0.46 | 0.88 |
| BMI<25, 25(OH)D<50 vs BMI<25, 25(OH)D≥50 | 0.8537 | -2.80, 4.50 | 0.52 | 0.92 |
| Neonatal lean mass index kg/m^2^ (R^2^ 0.13, R^2^_adj_ 0.12) | | | |  |
| Sex of infant (Ref: female) | 0.33 | 0.14, 0.52 | 0.0008 |  |
| Gestational age at birth, (wk) | 0.06 | -0.04, 0.16 | 0.24 |  |
| Infant age, (mo) | 0.56 | 0.09, 1.03 | 0.02 |  |
| UVB period (Ref: non-synthesizing period) | -0.12 | -0.31, 0.06 | 0.19 |  |
| Gestational weight gain, (kg) | -0.01 | -0.02, 0.01 | 0.34 |  |
| Maternal pre-pregnancy BMI (Ref: <25 kg/m^2^) | 0.22 | -0.02, 0.46 | 0.78 |  |
| Maternal 25(OH)D (Ref: ≥50 nmol/L) | 0.09 | -0.16, 0.35 | 0.35 |  |
| Maternal pre-pregnancy BMI*25(OH)D | | | 0.06 |  |
| BMI≥25, 25(OH)D<50 vs BMI≥25, 25(OH)D≥50 | -0.29 | -0.70, 0.12 | 0.07 | 0.26 |
| BMI≥25, 25(OH)D<50 vs BMI<25, 25(OH)D<50 | -0.16 | -0.59, 0.26 | 0.31 | 0.74 |
| BMI≥25, 25(OH)D<50 vs BMI<25, 25(OH)D≥50 | -0.07 | -0.44, 0.30 | 0.62 | 0.96 |
| BMI≥25, 25(OH)D≥50 vs BMI<25, 25(OH)D<50 | 0.13 | -0.27, 0.52 | 0.41 | 0.85 |
| BMI≥25, 25(OH)D≥50 vs BMI<25, 25(OH)D≥50 | 0.22 | -0.10, 0.54 | 0.08 | 0.29 |
| BMI<25, 25(OH)D<50 vs BMI<25, 25(OH)D≥50 | 0.09 | -0.25, 0.43 | 0.46 | 0.88 |
| ^1^Linear fixed effects model; ^2^Sex of infant (male vs. female); ^3^UVB period (April 1st-October 31^st^ or November 1st -March 31^st^); ^4^Maternal pre-pregnancy BMI (BMI<25 or BMI>25 kg/m^2^); ^5^maternal serum 25(OH)D (≥ or <50 nmol/L. Abbreviations: 25(OH)D: 25-hydroxyvitamin D; BMI: body mass index; UVB: ultraviolet B. | | | | |

**Supplemental Figures**

**Supplemental Figure 1** Infant sex differences in **A)** whole body fat mass **B)** whole body percentage fat mass, and **C)** fat mass index (FMI), **D)** whole body lean mass **E)** whole body percentage lean mass **F)** lean mass index (LMI). Data are mean ± SD; n= 83 male, n= 59 female. Data was compared using a linear fixed effects model for continuous variables (body composition) as outcome variables and sex as fixed effect, additional fixed factors included in the model were: gestational weight gain, gestational age (GA), UVB period at birth, actual age of infant at the postnatal visit, infant length and maternal pre-pregnancy BMI, 25(OH)D concentration and the interaction effect of last two variables followed by *post hoc* Tukey’s tests with Tukey-Kramer adjustment for multiple comparisons.

**Supplemental Figure 1** Sex dimorphism in neonatal body composition
